# Otosyphilis with Clinical and Serologic Responses with Weekly Intramuscular Penicillin: A Case Report and Literature Review

**DOI:** 10.1155/2022/3152383

**Published:** 2022-02-25

**Authors:** Ali Etemady-Deylamy, Getahun Abate

**Affiliations:** Division of Infectious Diseases, Saint Louis University, St. Louis, MO, USA

## Abstract

With the increasing prevalence of syphilis in different parts of the world, it is important to be cognizant of serious forms of syphilis. Otosyphilis is a rare form of syphilis with an extremely high rate of complications. Early diagnosis is essential to prevent a complete loss of hearing and decrease mortality. We present a unique case of otosyphilis who presented with left hearing loss, tinnitus, and vertigo, with audiometric findings confirming the hearing loss. An MRI brain showed asymmetric enhancement of the left cochlea and vestibular apparatus. She initially received oral steroids and trans-tympanic steroid injections with transient improvement of symptoms. She was diagnosed with syphilis through contact tracing after her ex-boyfriend tested positive. She received three weekly doses of intramuscular penicillin. Interestingly, her symptoms, rapid plasma regain (RPR) titer, and audiometry findings markedly improved. Once a diagnosis of otosyphilis was established, she received 14 days of intravenous penicillin.

## 1. Introduction

Syphilis, caused by *Treponema pallidum*, remains to be one of the major sexually transmitted infections in the USA [1, 2]. Despite available preventive and therapeutic strategies, more advanced disease forms occur [3]. Otosyphilis can occur at any stage of the disease, and it is suggested that patients with syphilis be routinely evaluated for otologic symptoms [4]. If not diagnosed early, otosyphilis may cause irreversible hearing loss and further progression of the disease [5].

## 2. Case Report

A thirty-three-year-old female patient presented with a decreased hearing of L-ear, tinnitus, and vertigo of three weeks' duration. She started to experience decreased hearing and tinnitus suddenly while she was playing the piano. She developed vertigo later the same day while she was exercising. She denied preceding trauma and having ear pain or ear discharge. There was no change in the severity of her symptoms. Her vital signs were stable. When she was seen at the ear, nose, and throat (ENT) clinic, she had no mastoid tenderness. Both ears had ear canals and tympanic membranes were intact with normal landmarks. An audiogram showed a normal right ear but a mild sensorineural hearing loss of 2000–8000 Hz in the left ear. Viral labyrinthitis and an early episode of endolymphatic hydrops were considered and started on a two-week course of oral steroid and a low salt diet and encouraged to hydrate. Her symptoms improved while she was on steroids, but only to recur when steroids were stopped.

**Figure 1:**
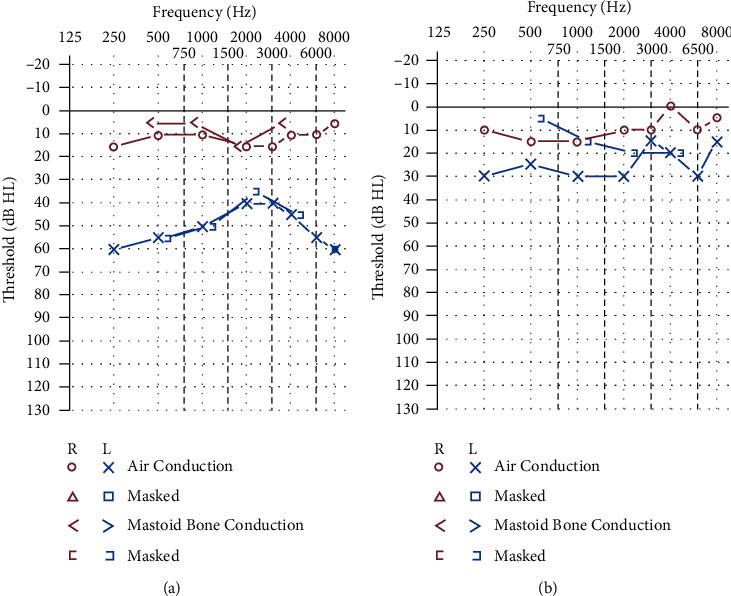
Audiogram changes after three weekly intramuscular doses of penicillin. (a) Before penicillin. (b) After penicillin.

Her course was complicated by the development of a generalized nonpruritic maculopapular rash as soon as she completed treatment with oral steroids. The rash spared the palms and soles. She had associated sore throat. She was evaluated by a dermatologist and received doxycycline (100 mg oral twice a day) for 4 weeks for possible folliculitis and rosacea. At the end of treatment with doxycycline, the skin rash resolved, but she continued to have decreased left ear hearing, tinnitus, and vertigo. Ear examination was unchanged and the left ear audiogram showed worsening (Figure 1(a)). MRI obtained while having vertigo showed asymmetric enhancement of the left cochlea and vestibular apparatus. About 2 months after her initial presentation, she received two trans-tympanic steroid injections (0.5 ml of 40 mg/ml Solu-Medrol) two weeks apart. Her left ear hearing transiently improved in noise and speech discrimination. Her symptoms continued to worsen.

About three months after presentation, she got a call from her former boyfriend who had been tested positive for syphilis and she was told to get tested. Subsequently, she was found to have a positive fluorescent treponemal antibody absorption (FTA-ABS) test, and a rapid plasma regain (RPR) titer of 1 : 64. The fourth-generation HIV ELISA was negative. She received three weekly doses of 2.4 million units of intramuscular penicillin G. Her L-ear symptoms markedly improved after the first penicillin dose and resolved after the third dose. A repeat audiogram showed a marked improvement (Figure 1(b)). The left ear exam showed a tympanic membrane with a small posterior perforation, and six weeks later, the exam showed the perforation was healing.

About 6 months after her presentation, she was seen at an infectious disease clinic. Tinnitus and vertigo have resolved. The left ear hearing was back to normal. A follow-up RPR titer was 1 : 8. Cerebrospinal fluid (CSF) analysis showed 2 white blood cells per *μ*L with normal glucose and protein. CSF venerable disease research laboratory test (VDRL) was negative. Although her clinical symptoms resolved and RPR titer decreased, the audiogram result of the left ear was not as normal as the right ear. Therefore, she was started on a 14-day course of intravenous penicillin (24 million units per day).

## 3. Discussion

This case is unique because (i) the diagnosis of otosyphilis was made after a patient was identified through contact tracing, (ii) partial or no response to steroids or doxycycline, and (iii) marked clinical, audiometric, and serologic response following three doses of weekly intramuscular penicillin G. The prevalence of syphilis is increasing, mainly associated with HIV [2, 6]. This calls for renewed emphasis on early detection to prevent long-term sequelae.

Otosyphilis is one of the complications of syphilis that may lead to deafness and other complications if untreated [4]. Otosyphilis can occur at any stage of syphilis [7]. The diagnosis of otosyphilis is challenging because presenting clinical symptoms are nonspecific and could be seen in other conditions, including labyrinthitis, vestibular neuritis, Meniere's disease, acoustic neuroma, autoimmune disease, and stroke [4, 8, 9]. Unilateral or bilateral hearing loss, tinnitus, and vertigo which our patient had are the common manifestations of otosyphilis [7, 9–24]. In some patients, MRI brain could be normal [15, 19, 23, 24]. Reported MRI abnormalities associated with otosyphilis include meningeal enhancement [13], cochlear enhancement [20], intracanalicular mass [20], auditory polyneuritis [21], and periventricular and deep white matter lesions [12]. The MRI in our patient showed asymmetric enhancement of the left cochlea and vestibular apparatus. To our knowledge, enhancement of the vestibular apparatus was not reported in association with otosyphilis before. Furthermore, audiometry may detect nonspecific abnormalities. As seen in our patient, the severity of sensorineural hearing loss may worsen without appropriate treatment. In these cases, the FTA-ABS test and RPR should be obtained as soon as possible. Because otosyphilis is considered as neurosyphilis, CSF analysis is part of the workup. CSF findings could be normal [11] or may show elevated WBC [10, 13, 20, 21, 23], elevated protein [9, 20, 21, 23], and a positive VDRL [9, 10, 12–14]. In our patient, lumbar puncture was done after intramuscular penicillin and CSF showed no abnormality.

Because it is rare, treatment guidelines rely on results from case series and case reports [25]. It is recommended that syphilis of sensory organs such as the ear and eye be treated the same way as neurosyphilis with intravenous penicillin G for 10–14 days [25]. Intravenous penicillin for 10–14 days had shown subjective clinical improvement in 34/55 (62%) of cases [10–12] and audiometric improvement in 18/44 (41%) of reported cases who had audiogram [10, 11, 17, 21]. The response to weekly intramuscular penicillin appears much lower than the response seen following intravenous penicillin treatment. Intramuscular penicillin failed to provide symptomatic relief in 8/8 (100%) HIV-positive patients who presented with hearing loss and tinnitus [13]. Six of these 8 patients had meningeal enhancement on MRI, indicating that the treatment for these patients with neurosyphilis/otosyphilis may have been suboptimal. In another study, 17 patients with unknown HIV status received intramuscular penicillin for more than 12 weeks; transient clinical improvement was seen only in 29% of cases, and audiogram improvement in 18% [15]. Interestingly, our patient showed marked clinical, laboratory, and audiometric improvement after three weekly doses of intramuscular penicillin. Because of the risk of recurrence [15] and limited success in reported cases where intramuscular penicillin was used, our patient received additional treatment with intravenous penicillin for 14 days.

In conclusion, otosyphilis should be considered in sexually active patients with unilateral sensorineural hearing loss, and appropriate treatment is essential to prevent irreversible hearing loss.

## Data Availability

This is a case report and access to additional patient data is restricted.
